# A study on the effects of virtual gaming environment stimuli on adolescents' environmental awareness and behavior: the mediating role of environmental trust

**DOI:** 10.3389/fpsyg.2025.1716373

**Published:** 2025-11-24

**Authors:** Xinyu Hu, Chenyu Wu, Le Wang

**Affiliations:** 1 College of Fashion and Design, Donghua University, Shanghai, China; 2 Glory Sun School of Business and Management, Donghua University, Shanghai, China

**Keywords:** virtual games, environmental trust, adolescent psychology, eco-friendly behavior, S-O-R theory

## Abstract

**Purpose:**

Motivating pro-environmental behavior among adolescents remains an ongoing challenge, and commercial digital games offer an emerging avenue to address this. Despite the immense potential of games, the underlying psychological mechanisms that translate virtual environmental stimuli into real-world actions remain poorly understood. This study constructs a model to examine these conversion pathways, thereby providing clear insights and empirical support for designing effective environmental interventions using mainstream gaming platforms.

**Methods:**

Based on questionnaire data from 2,279 Chinese adolescent gamers of the popular title Honor of Kings, this study constructs a parallel chain mediation model following the Stimulus-Organism-Response framework. Through Pearson correlation analysis, Heterotrait–Monotrait ratio (HTMT) of correlations, and the Bootstrap method using the SPSS PROCESS macro (Model 6), we systematically examined the causal pathways from game stimuli to pro-environmental behavior. This validated a chained mediation mechanism centered on Game Appraisal and Emotional Response, Pro-Environmental Trust, and Pro-Environmental Intention.

**Results:**

The findings clearly reveal a complete psychological conversion pathway. First, in-game environmental stimuli, such as eco-themed Character Outfits and Environmental Game Mechanisms, significantly and positively predict players' Game Appraisal and Emotional Response. This emotional and cognitive engagement, in turn, robustly fosters Pro-Environmental Trust in the game platform. The analysis confirms that this trust acts as a pivotal mediator, significantly influencing two distinct behavioral intentions: the Intention to Purchase Environmental Products and the Intention to Spread Environmental Messages on social media. Ultimately, both of these intentions were found to be significant positive predictors of final Pro-Environmental Behavior.

**Conclusions:**

This study confirms that Pro-Environmental Trust serves as a crucial psychological bridge converting players' Game Appraisal and Emotional Response into real-world pro-environmental intentions and ultimately, Pro-Environmental Behavior. By elucidating this conversion mechanism, the research contributes to existing literature. This finding provides clear implications for game developers and policymakers: to effectively leverage games for environmental promotion, fostering genuine player trust through authentic environmental initiatives is equally important as designing immersive content.

## 1 Introduction

As global climate change and ecological issues grow increasingly severe, environmental protection has become a critical issue for human societal development. Enhancing public environmental awareness and promoting sustainable behaviors are particularly urgent (Zeng et al., 2020). As the future backbone of society, adolescents' Pro-Environmental Awareness and Habits (PEAH) not only shape their personal growth but also profoundly impact the overall progress of ecological civilization (Zibenberg et al., 2018). However, a notable gap persists between the high level of environmental awareness among young people and their actual engagement in consistent pro-environmental behaviors. For instance, while a recent survey indicates that 87.11% of young respondents recognize the urgency of global environmental issues, and 78.68% report saving energy in daily life, participation drops in other key areas—such as reducing takeout (less commonly practiced) and consistently sorting waste—highlighting a disconnect between attitude and action (Zhongguo Qingnian Bao and Zhongqing Xiaomei, 2023). This prominent attitude-behavior gap highlights the urgent need for more effective intervention strategies. Research indicates that adolescence is a critical period for the formation of values and behavioral patterns, where effective environmental education can lay the foundation for long-term eco-friendly behaviors (Wang and Tang, 2023). Consequently, exploring effective pathways to enhance adolescents' PEAH has become a significant research focus in environmental education and social psychology (Coopwood, 2008).

Virtual Environmental Stimuli (VES) play a significant role in individual cognitive development. External information inputs—such as visual, auditory, and situational simulations—can substantially influence attitudes and perceptions toward the environment (Cocquyt and Palombo, 2023; Ophir et al., 2009). Among adolescents specifically, diverse environmental stimuli facilitate cognitive optimization and behavioral intention transformation (Polisetty et al., 2024). With the advancement of digital technology, VES has emerged as a novel medium demonstrating unique potential in education and socialization. Through immersive experiences and interactive mechanisms, it simulates real-world scenarios to facilitate knowledge acquisition and cognitive construction (Pedrouzo and Krynski, 2023).

Taking the widely popular Chinese multiplayer online battle arena game Honor of Kings as an example, this game not only enjoys extremely high popularity and influence among young people-with reports indicating over 100 million daily active users, a substantial portion of whom are adolescents (Ye et al., 2022), but also incorporates various environmental protection-related visual and narrative elements into its design (Tencent Games, 2017). For instance, skin designs, scene layouts, and limited-time events frequently feature themes of natural ecosystems, green energy, or sustainable development, subtly conveying environmental protection concepts (Shahzad et al., 2023; Bassanelli et al., 2025). Such VES may potentially influence players' PEAH. Pro-Environmental Awareness (PEA) refers to an individual's knowledge, attitudes, and concern regarding environmental issues, while pro-environmental behavior encompasses specific actions such as energy conservation, emission reduction, and waste sorting (Dönmez and Yardimci, 2024). Research indicates that PEA is a key antecedent variable in predicting pro-environmental behavior, with higher levels of PEA often promoting actual environmental actions (Demir et al., 2021). Previous research further confirms that enhancing PEA through media interventions can, to a certain extent, boost individuals' enthusiasm for participating in environmental protection. Virtual game environments are emerging as a significant intervention vehicle in this regard (Linge et al., 2024).

Pro-Environmental Behavior (PEB) is defined as conscious actions taken by individuals to minimize their negative impact on the natural world. These behaviors span a wide spectrum, from private-sphere actions like recycling, reducing water consumption, and purchasing sustainable products, to public-sphere actions such as environmental activism and advocating for green policies (Tian and Liu, 2022). Extensive research has identified key antecedents of PEB, which are often categorized into internal and external factors. Internal factors include psychological variables such as environmental knowledge, personal values, attitudes, and a sense of personal responsibility. For instance, studies have demonstrated that when environmental knowledge is combined with trust, it can significantly predict the purchase intention for eco-friendly products. External factors, on the other hand, encompass social influences like community norms, policy incentives, and media exposure (Tam, 2025; Zelenski and Desrochers, 2021).

Despite this substantial body of research, a significant gap remains in understanding how modern digital media, particularly mainstream commercial video games, influence PEB. Most existing studies focus on traditional media or direct educational interventions with limited reach, yet the indirect and immersive influence of commercial video games—which engage hundreds of millions of adolescents daily—remains virtually unexamined (Pallavicini et al., 2022). The novelty of our study lies precisely here: first, it moves beyond purpose-built educational tools to investigate a globally popular commercial game, examining how environmental themes embedded in entertainment can trigger pro-environmental action on a massive scale. Second, and more importantly, it proposes and empirically tests a novel psychological pathway. Instead of merely assessing awareness, our research delves into the deeper mechanism of how in-game experiences are converted into real-world behavior, identifying Pro-Environmental Trust (PET) as the critical, yet previously overlooked, psychological bridge that links virtual engagement to tangible pro-environmental intentions and actions.

Pro-Environmental Trust (PET) refers to an individual's confidence in and recognition of the environmental protection measures taken by entities such as governments, businesses, and social organizations (Asif et al., 2023). Previous research indicates that PET may play a crucial mediating role between environmental cognition and eco-friendly behavior (Li et al., 2021). High levels of PET enhance individuals' receptivity to environmental information, increase their willingness to engage in eco-friendly actions, and mitigate the “intention-behavior gap” between cognition and action (Wang, 2024). For instance, studies indicate that individuals are more likely to translate PEA into action when they perceive environmental policies or public initiatives as trustworthy (Yang et al., 2022). Furthermore, PET has been found to moderate the impact of external stimuli on individuals' environmental attitudes and behaviors (Mathers-Jones and Todd, 2023). However, the mediating mechanism of PET between VES and adolescents' PEAH remains under-explored, providing a theoretical entry point for this study.

Although existing research has examined the effects of environmental education, media communication, and gamified learning on adolescents' PEAH, most studies have focused on traditional educational settings or specially designed serious games. Research on the mechanisms underlying the impact of environmental stimuli within mainstream commercial virtual gaming environments remains insufficient (Godfrey et al., 2025; Marques et al., 2023) especially given the vast and deeply engaged adolescent audiences these platforms command. Specifically, the role of PET as a potential mediating variable in virtual gaming contexts has yet to be empirically tested. Therefore, this study uses Honor of Kings as a case to examine the impact of VES on adolescents' PEAH and explore the mediating role of PET. The significance of this research lies in: first, expanding the theoretical boundaries of environmental education and media effects studies by incorporating virtual games as an emerging medium into the analytical framework; second, revealing the mediating mechanism of PET to provide empirical evidence for designing more effective intervention strategies, thereby promoting the development of Pro-Environmental Behavior (PEB). This study aims to employ quantitative analytical methods to examine the influence pathways of VES on adolescents' PEAH and validate the mediating effect of PET within this relationship. This research seeks to fill existing gaps in the literature and advance both theoretical understanding and practical applications.

## 2 Theory and hypotheses

### 2.1 Theoretical foundation: the S-O-R theory

This study is grounded in the **Stimulus-Organism-Response (S-O-R)** framework, a foundational model in environmental psychology and behavioral studies that effectively explains how external environmental cues influence internal psychological processes, ultimately leading to specific behaviors (Wu and Santana, 2022; Lu et al., 2025). The model posits that external stimuli (S) trigger internal affective and cognitive states within the individual (the Organism, O), which in turn drive behavioral responses (R).

We selected the S-O-R framework over alternative models like the Theory of Planned Behavior (TPB) or models focusing solely on Normative Social Influence (NSI) for its superior ability to integrate both emotional and cognitive pathways (Janakiraman et al., 2021; Zhu et al., 2024). Given that commercial video games like *Honor of Kings* are immersive, experience-heavy environments designed to elicit strong emotional engagement, the S-O-R model provides greater theoretical explanatory power for analyzing how such virtual experiences stimulate players' intrinsic motivation and catalyze behavioral change.

Within our research model, the S-O-R constructs are operationalized as follows:

- **Stimulus (S):** This encompasses the Virtual Environmental Stimuli (VES) embedded within the game, specifically the Character Outfits (CO) with eco-friendly themes and the Environmental Game Mechanisms (EGM) that promote pro-environmental interactions.
- **Organism (O):** This represents the internal psychological mechanisms of the player. It is conceptualized as a sequential process, beginning with the immediate Game Appraisal and Emotional Response (GAER) to the stimuli, which then fosters a more stable, cognitive evaluation of the platform's credibility, termed Pro-Environmental Trust (PET).
- **Response (R):** This signifies the ultimate behavioral outcome, which is the real-world Pro-Environmental Behavior (PEB). This final response is preceded by the formation of specific behavioral intentions, namely the Intention to Purchase Environmental Products (ITP) and the Intention to Spread Environmental Messages (ITS).

By applying this framework, our study aims to validate the complete psychological pathway from in-game stimuli to real-world action, with a specific focus on examining the pivotal mediating roles of GAER and PET.

### 2.2 Hypothesis development

#### 2.2.1 VES and GAER

This paper operationalizes the core psychological mechanisms of players in eco-friendly games as GAER (Game Appraisal and Emotional Response), serving as a bridge between external stimuli and subjective reactions. GAER comprises two dimensions: (1) Cognitive Appraisal, referring to players' understanding and logical endorsement of in-game environmental concepts and cues (Fernandes et al., 2024). (2) Emotional Response, denoting the comprehensive affective experience—such as immersion, resonance, and belonging—triggered by environmental content (Zhang et al., 2021).

**Figure 1:**
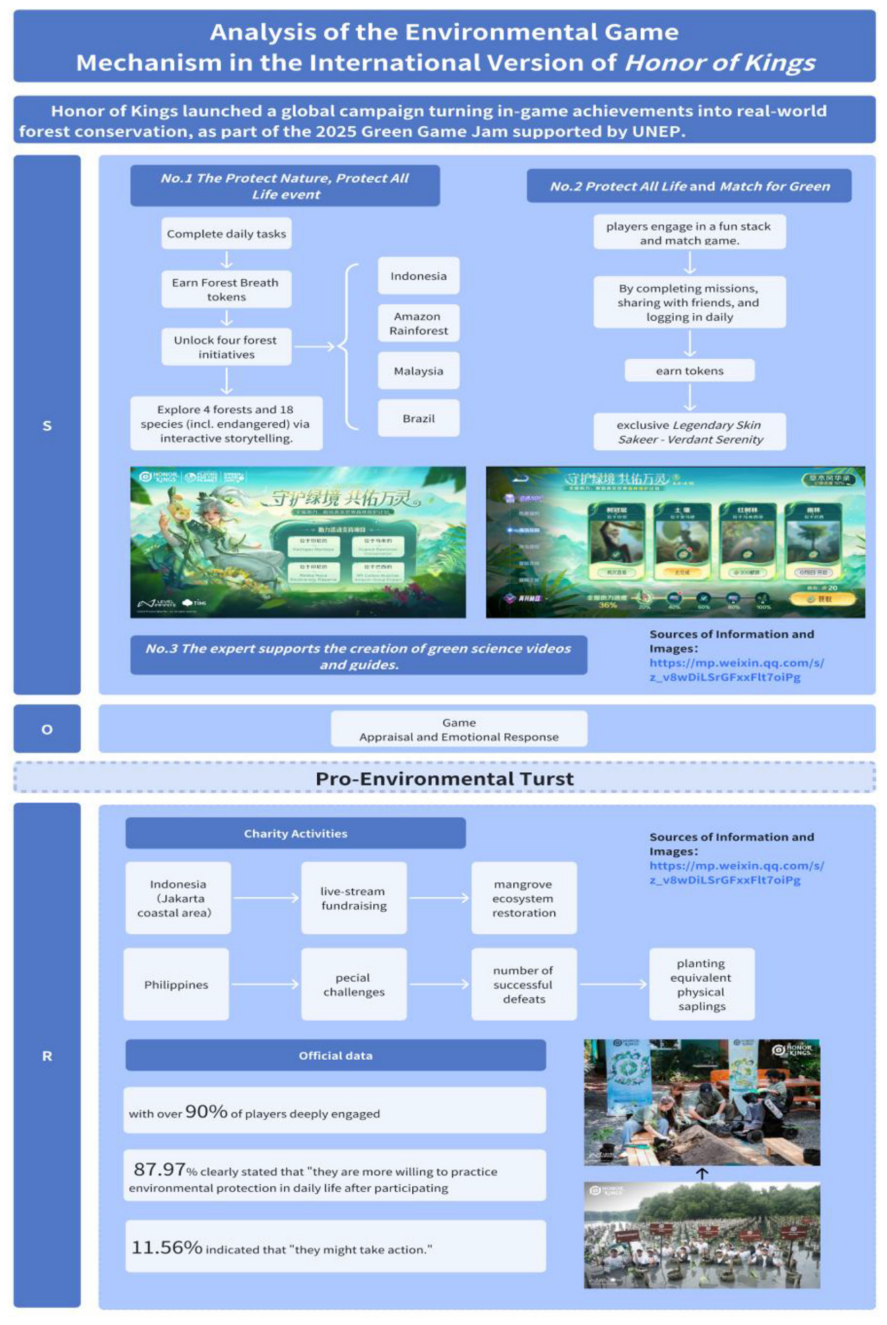
Honor of kings environmental game mechanics.

VES manifests in diverse forms. Taking Honor of Kings as an example, it systematically constructs cultural imagery of human-nature harmony by integrating natural elements and the Daoist philosophy of “following nature's way” into the character designs of figures like Zhuang Zhou and Guiguzi (Anderson et al., 2025). As shown in Figure 1, its international server collaborated with the UN Green Game Jam competition to launch a limited-time Earth Day event. Through environmental mini-games, educational videos, and special rewards—collectively termed EGM—it blended entertainment with environmental education. These designs created powerful external stimuli. Official data indicates the event attracted deep engagement from over 90% of players (Level Infinite, n.d.).

Research indicates that compared to traditional one-way advocacy, the interactive environment of virtual games more effectively stimulates environmental awareness (Khelifa and Mahdjoub, 2021). The interactivity, achievement rewards, and community features provided by games transform players from passive information recipients into active participants and disseminators of PEB, significantly enhancing the contextual relevance and real-world connection of environmental concepts (Razali et al., 2022). Empirical research confirms that effective external environmental stimuli activate individuals' cognitive processes and emotional resonance, promoting the internalization of environmental values (Shao and Liu, 2022; Chen et al., 2025). Based on the above theoretical and empirical foundations, this paper proposes the following hypotheses:

**H1a: CO positively influences GAER**.

**H1b: EGM positively influences GAER**.

#### 2.2.2 The Mediating Role of PET

This study introduces PET as a key mediating variable, which refers to players' overall trust in a gaming platform's environmental commitments, image, and actions (Xing et al., 2022). Existing research has established that Pro-Environmental Trust (PET) serves as a critical bridge, significantly enhancing users' Pro-Environmental Intention (PEI), which encompasses both the Intention to Purchase Environmental Products (ITP) and the Intention to Spread Environmental Messages on Social Media (ITS; Asif et al., 2023; Irawan and Benius, 2022).

Within this study's model, PET assumes a dual role. First, it serves as the core mechanism transforming GAER into stable behavioral intentions. The relationship between GAER and PET represents a shift from immediate reactions to specific stimuli toward stable attitudes toward the platform (Chen et al., 2023). Specifically, GAER reflects players' immediate, situational cognitive and emotional responses triggered by individual COs or EGMs, while PET signifies the long-term accumulation of these positive experiences, ultimately forming trust in the entire platform (Hawrysz et al., 2024). This stems from players' perception of the platform's sustained commitment to environmental responsibility through continuous participation in eco-themed activities, use of eco-friendly COs, and engagement with the platform's environmental initiatives (Lee and Jeong, 2022).

Second, PET's function extends beyond this, serving as the critical bridge facilitating the migration of Pro-Environmental Intention (PEI) from the virtual to the real world. Research indicates that when adolescent players develop high trust in the platform's environmental commitments, this trust strengthens their identity and motivates them to seek behavioral consistency in real life. This effectively transforms PEI, which emerges in the virtual space, into observable PEB in the real world, ultimately achieving unity between knowledge and action in environmental concepts. Consequently, this paper proposes the following hypothesis:

**H2: GAER directly and positively influences players' PEI**.

**H3: PET positively influences players' PEI**.

**H-Mid1: GAER indirectly and positively influences ITP through PET**.

**H-Mid2: GAER indirectly and positively influences ITS through PET**.

**H4: PEI positively influences players' PEB performance**.

In summary, this study proposes a novel research framework by constructing a chain-mediated transmission model involving GAER and PET, and verifying its impact on players' real-world PEB. This contributes to a deeper understanding of VES and how digital gaming experiences can effectively foster environmental responsibility among adolescents.

## 3 Methods

### 3.1 Participants

This study employed convenience sampling to conduct an online and offline questionnaire survey targeting active adolescent players of Honor of Kings, ultimately yielding 2,279 valid samples (Stratton, 2021). Data collection occurred from April to August 2025. Online distribution utilized mainstream social platforms and player communities, while offline distribution took place at university campuses and esports events to ensure sample diversity. To guarantee data quality, the study implemented quality control measures including response incentives and false information screening, achieving a high valid response rate of 98.96%. The sample predominantly comprised males (59.1%), with a polarized educational distribution centered on elementary school students (33.9%) and college students (52.2%). Over half of the players (51.1%) resided in third- and fourth-tier cities or below, and most came from families with strong educational backgrounds (80.7% had parents with higher education). Regarding gaming investment, the sample exhibited deep engagement: over 60% (66.0%) of players spent more than 7 h per week gaming, and the majority (60.1%) had established stable payment habits, with monthly spending concentrated in the 200–500-yuan range.

### 3.2 Measures

This study employed seven scales to systematically evaluate VES (CO, EGM), player psychological responses (GAER, PET), and behavioral conversion outcomes (ITP, ITS, PEB). All scales were adapted from environmental psychology (Sokolova et al., 2023; Ischen et al., 2022; Ellen et al., 1991); the field of game studies and consumer behavior studies (Zaichkowsky, 1994; Taufique et al., 2019; Chen, 2010), and PEB (Chen and Chang, 2012; Chu and Kim, 2011; Rosli et al., 2024). They were adapted for the gaming context of Honor of Kings.

The scales underwent rigorous optimization. To enhance model fit, items with ambiguous wording or low relevance to gaming scenarios were removed following pre-review and pilot testing. The final formal questionnaire comprises 24 measurement items: CO (3 items), EGM (3 items), GAER (5 items), PET (3 items), ITP (3 items), ITS (3 items), and PEB (4 items), as detailed in Table 1.

**Table 1:** The reliability and validity testing.

**Variable**	**Items**	**Item number**	**α**	**Loading**	**CR**	**AVE**
CO	This outfit projects an eco-friendly image.	CO1	0.918	0.801	0.837	0.632
	This outfit communicates pro-environmental values.	CO2		0.780		
	The outfit's environmental messaging is clearly visible.	CO3		0.803		
EGM	My individual actions make a difference in these green mechanisms.	EGM1	0.883	0.818	0.801	0.574
	Individual actions collectively contribute to environmental improvement.	EGM2		0.719		
	I can influence other players' engagement with green mechanisms.	EGM3		0.733		
GAER	I clearly noticed environmental cues during gameplay.	GAER1	0.911	0.744	0.849	0.53
	I understand the meaning behind these green elements.	GAER2		0.699		
	The game's environmental themes are important to me.	GAER3		0.747		
	These green elements enhance my connection with nature.	GAER4		0.710		
	These green elements are emotionally resonant.	GAER5		0.738		
PET	The game's environmental information is trustworthy.	PET1	0.863	0.742	0.775	0.535
	The game operator is reliable in environmental matters.	PET2		0.698		
	Overall, I trust its environmental performance.	PET3		0.754		
ITP	I prioritize eco-friendly products when available.	ITP1	0.872	0.704	0.769	0.527
	I prefer environmentally sustainable options at equal price.	ITP2		0.765		
	I am willing to pay for environmental attributes.	ITP3		0.707		
ITS	I actively share environmental views on social media.	ITS1	0.893	0.764	0.802	0.574
	I share valuable green content with others.	ITS2		0.764		
	I plan to share environmental content monthly.	ITS3		0.745		
PEB	I prioritize eco-friendly products.	EB1	0.934	0.744	0.837	0.563
	I make environmentally conscious shopping decisions.	EB2		0.769		
	I actively sort waste for recycling.	EB3		0.766		
	I proactively share environmental knowledge.	EB4		0.721		

Among these, the final dependent variable of this study—Real-world PEB—is defined as voluntary, environmentally beneficial actions taken by players outside their gaming experience. Examples include systematic waste sorting habits, conscious reduction in single-use product consumption, and proactive sharing of environmental knowledge.

To clarify the theoretical progression in our model, it is crucial to differentiate the nature of its final variables. ITP and ITS are operationalized as specific, forward-looking intentions. They represent a stated willingness to perform particular actions in the future within the domains of consumption and social communication. In contrast, the final dependent variable, PEB (Pro-Environmental Behavior), serves as a broader measure of established, recurring behaviors that respondents already engage in. This is substantiated by scale items that inquire about concrete, routine actions (e.g., ‘I actively sort waste for recycling'). Therefore, our model's logic is not to test how intention predicts intention, but rather to examine how specific, future-oriented intentions, fostered by the gaming experience, contribute to a general pattern of existing, real-world environmental action.

First, the data adequacy analysis yielded favorable results, with a KMO value of 0.960 (>0.7) and a significant Bartlett's sphericity test (*p* < 0.001), indicating the data are highly suitable for factor analysis. To validate the scale's reliability, we calculated Cronbach's α for each construct. As shown in Table 1, all α values significantly exceeded the 0.7 standard, demonstrating good internal consistency. To assess construct validity, we calculated the Composite Reliability (CR) for each construct. As shown in Table 1, all CR values exceeded 0.7, indicating strong construct validity for this scale. To evaluate convergent validity, we computed the Average Variance Extracted (AVE) for each construct. All AVE values exceeded 0.5, confirming strong convergent validity for this scale.

### 3.3 Data analysis

This study employed IBM SPSS Statistics 26.0 and the SPSS macro-PROCESS for data analysis. Descriptive statistics were first conducted on 2,279 valid samples, followed by KMO and Bartlett's sphericity tests to validate data suitability. Subsequently, reliability and validity tests were conducted: Cronbach's α coefficients were calculated for each construct to assess reliability; convergent validity was examined through factor loadings, CR, and AVE; discriminant validity was evaluated using the Heterotrait–Monotrait ratio (HTMT) of correlations. Means, standard deviations, and Pearson correlation matrices were then computed for each variable. Finally, PROCESS Model 6 was employed to examine the chained mediating effects of independent variables (CO, EGM) on the dependent variable (PEB) through GAER, PET, ITS, and ITP, using Bootstrap sampling (5,000 iterations). All tests were conducted at a significance level of α=0.05, with two-tailed *p* < 0.05 considered statistically significant.

## 4 Results

This chapter presents a detailed account of the findings from the data analysis. Firstly, we examined the reliability and validity of the measurement model, with particular emphasis on assessing discriminant validity using the more stringent HTMT criteria. Subsequently, the results of descriptive statistics and correlation analyses are presented. Finally, the outcomes of the multiple regression and chained mediation analyses employed to test the core hypotheses of this study are reported.

### 4.1 Discriminant validity analysis

To rigorously assess the discriminant validity of the constructs, we employed the HTMT criterion, as recommended by Henseler et al. (2015). This method is considered more reliable than the traditional Fornell–Larcker criterion. The results of the HTMT analysis are presented in Table 2.

**Table 2:** HTMT results for discriminant validity.

**Variable**	**CO**	**EGM**	**GAER**	**PET**	**ITP**	**ITS**	**PEB**
CO	1.00						
EGM	0.23	1.00					
GAER	0.64	0.73	1.00				
PET	0.73	0.68	0.72	1.00			
ITP	0.47	0.88	0.65	0.70	1.00		
ITS	0.75	0.64	0.64	0.67	0.71	1.00	
PEB	0.66	0.74	0.54	0.63	0.72	0.69	1.00

As shown in Table 2, all HTMT values ranged from 0.71 to 0.83. These values are well below the conservative threshold of 0.85, indicating that the constructs are distinct from each other. Furthermore, the bootstrapping confidence intervals (5,000 samples) for all HTMT values had an upper limit below 1.00. These results provide strong evidence of discriminant validity among the seven constructs in our measurement model, ensuring that they measure conceptually distinct phenomena.

### 4.2 Descriptive statistics and correlations

The means, standard deviations, and Pearson correlation coefficient matrix for each core variable in this study are presented in Table 3. Descriptive statistics indicate that the means for all variables fall within the upper-middle range, suggesting that the sample players perceive in-game environmental design positively and tend to exhibit favorable psychological responses and behavioral intentions.

**Table 3:** Means, standard deviations, and correlations.

**Variable**	***M***	**SD**	**1**	**2**	**3**	**4**	**5**	**6**	**7**
CO	3.08	1.28	1						
EGM	3.42	1.25	0.21	1					
GAER	3.21	1.01	0.58	0.59	1				
PET	3.23	1.05	0.55	0.52	0.58	1			
ITP	3.16	1.04	0.53	0.51	0.59	0.56	1		
ITS	3.22	1.06	0.52	0.52	0.58	0.57	0.55	1	
PEB	3.16	1.05	0.51	0.49	0.57	0.58	0.59	0.54	1

Correlation analysis clearly demonstrates significant positive correlations among all core constructs in this study (all *p*-values < 0.001). Specifically, CO, EGM, GAER, and PET exhibit strong positive correlations, preliminarily validating the direct influence of “S” on “O” within the S-O-R model. Simultaneously, the core psychological mechanism variables (GAER, PET) also exhibit high positive correlations with the final behavioral conversion outcomes (ITP, ITS, PEB). These results provide robust data support for subsequent mediation effect testing and preliminarily outline the variable relationship pathways within this study's theoretical model.

### 4.3 Mediation analysis

**Figure 2:**
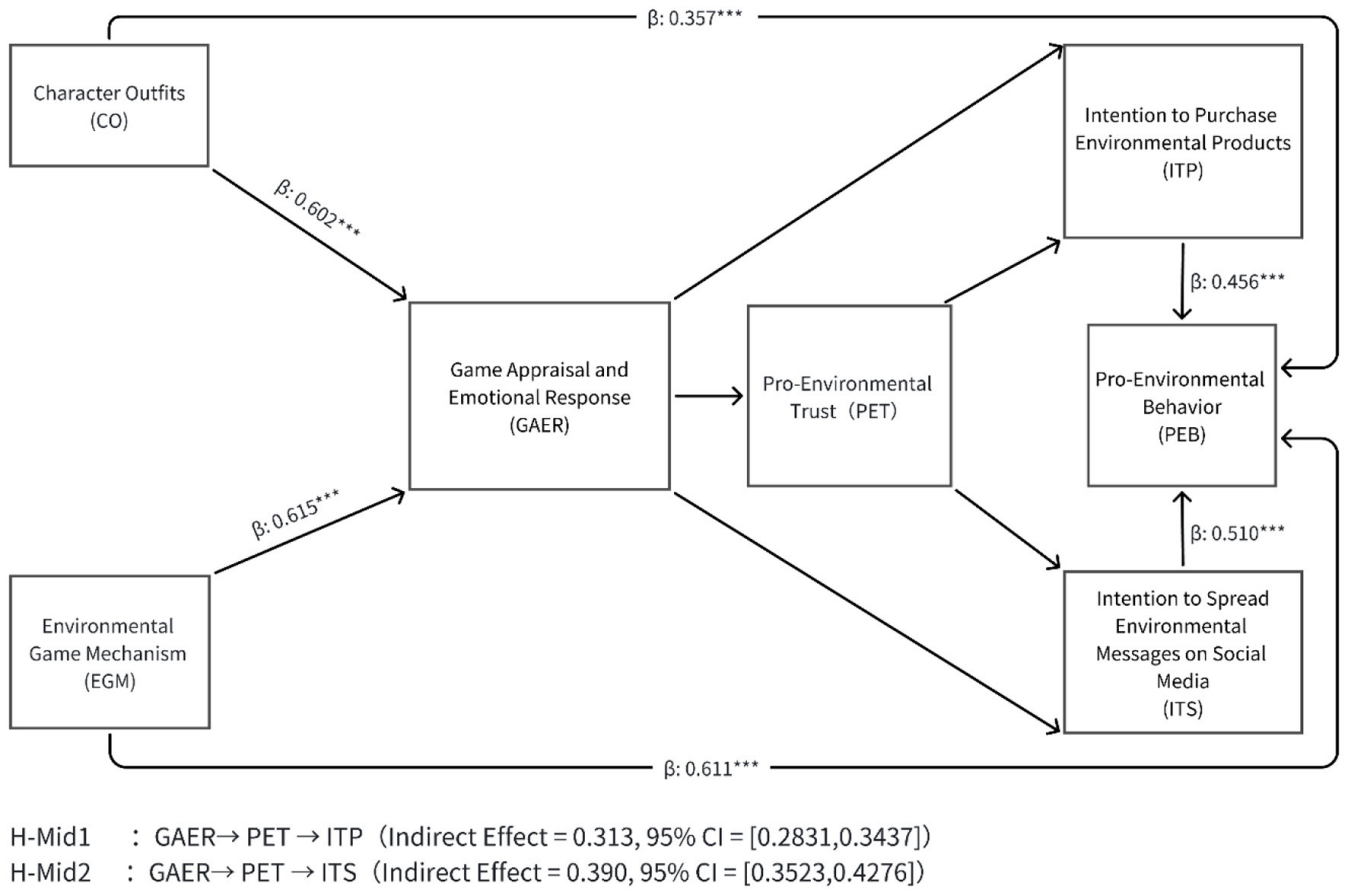
The research model with path coefficients.

This study aims to examine a parallel chain mediation model, specifically investigating the complex mechanism whereby CO and EGM ultimately influence PEB through GAER and PET, and separately through ITS and ITP, as illustrated in Figure 2. To this end, the Model 6 process model developed by Hayes (2013) within the SPSS Process macro was employed for testing. Bootstrap sampling was conducted with 5,000 iterations at a 95% confidence level. The criterion for significance was set as follows: if the 95% confidence interval of a standardized path coefficient did not include zero, the effect was deemed significant.

Prior to conducting chained mediation analysis, this study first performed basic regression tests on each path segment of the model. Results showed (see Tables 4, 5) that in the front-end paths of the model, both CO (β = 0.602, *p* < 0.001) and EGM (β = 0.615, *p* < 0.001) significantly and positively predicted GAER; In the back-end paths, ITP (β = 0.456, *p* < 0.001) and ITS (β = 0.510, *p* < 0.001) also significantly and positively predicted PEB. All key path coefficients were significantly positive, providing preliminary support for chain mediation testing. The results of chain mediation effect testing (see Tables 6, 7) indicate that GAER has a significant total effect on PEB (Total Effect = 0.903, 95% CI [0.8822, 0.9242]). Specific mediation path analyses are as follows:

- Paths with ITP as the mediating variable (Table 4):

Path 1 (partial mediation): GAER → ITP → PEB, with an indirect effect value of 0.151, 95% CI [0.1304, 0.1751].

Path 2 (partial mediation): GAER → PET → PEB, indirect effect value 0.262, 95% CI [0.2184, 0.3061].

Path 3 (Chain mediation): GAER → PET → ITP → PEB, indirect effect value 0.313, 95% CI [0.2831, 0.3437].

- Paths with ITS as mediating variable (Table 6):

Path 4 (partial mediation): GAER → ITS → PEB, indirect effect value 0.149, 95% CI [0.1269, 0.1718].

Path 5 (partial mediation): GAER → PET → PEB, with an indirect effect value of 0.207, 95% CI [0.1590, 0.2529].

Path 6 (Chain Mediation): GAER → PET → ITS → PEB, with an indirect effect value of 0.390, 95% CI [0.3523, 0.4276].

**Table 4:** Results of multiple regression analysis of CO and EGM on GAER.

**Variable**	***B***	**SE**	**β**	***t***	***p***	**95% CI**
Constant	0.054	0.024		2.25	0.023	[0.007, 0.100]
CO	0.474	0.005	0.602	94.80	< 0.001	[0.464, 0.485]
EGM	0.495	0.006	0.615	82.50	< 0.001	[0.484, 0.506]

**Table 5:** Predicting PEB from ITP and ITS.

**Variable**	***B***	**SE**	**β**	***t***	***p***	**95% CI**
Constant	0.090	0.024		3.75	< 0.001	[0.007, 0.100]
CO	0.460	0.016	0.456	28.75	< 0.001	[0.464, 0.485]
EGM	0.504	0.016	0.510	31.50	< 0.001	[0.484, 0.506]

**Table 6:** Mediation analysis for ITP.

**Model**	**Effect**	**Boot SE**	**Boot 95%CI**
Total effect	0.903	0.011	[0.882, 0.924]
Direct effect	0.149	0.021	[0.108, 0.190]
Total indirect effects	0.754	0.020	[0.714, 0.793]
GAER → ITP → PEB	0.151	0.011	[0.130, 0.175]
GAER → PET → PEB	0.262	0.022	[0.218, 0.306]
GAER → PET → ITP → PEB	0.313	0.016	[0.283, 0.344]

^*^Effects are unstandardized coefficients. Bootstrap sample size = 5,000.

**Table 7:** Mediation analysis for ITS.

**Model**	**Effect**	**Boot SE**	**Boot 95%CI**
Total effect	0.903	0.011	[0.882, 0.924]
Direct effect	0.157	0.021	[0.117, 0.198]
Total indirect effects	0.746	0.020	[0.706, 0.785]
GAER → ITS → PEB	0.149	0.012	[0.127, 0.172]
GAER → PET → PEB	0.207	0.024	[0.159, 0.253]
GAER → PET → ITS → PEB	0.390	0.019	[0.352, 0.428]

^*^Effects are unstandardized coefficients. Bootstrap sample size = 5,000.

Since the Bootstrap confidence intervals for all mediating paths above do not include zero, they are all statistically significant. The results demonstrate that GAER not only directly influences PEB but also generates multiple indirect effect paths through PET, ITP, and ITS. Among these, the chained path “GAER → PET → ITS → PEB” exhibits the largest effect size (0.390).

This study further examined the direct effect of the independent variable (S) on the dependent variable (R) within the S-O-R framework to construct a more comprehensive model and compare direct effects with mediating effects. We employed the SPSS PROCESS macro (Model 4), incorporating all mediating variables (GAER, PET, ITS, ITP) into the regression equation while controlling for covariates (CO or EGM), to examine the direct effects of CO and EGM on PEB.

Results are presented in Table 8. The total effect of CO on PEB was significant (β = 0.3574, *p* < 0.001). However, after incorporating all mediating variables into the model, the direct effect of CO on PEB became non-significant (β = −0.0093, *p* = 0.427).

**Table 8:** Summary of total, direct, and indirect effects.

***X***	**Path**	**Effect type**	**β**	**SE**	***P***	**95% CI**
CO	CO → PEB	Total effect	0.3574	0.0054	< 0.001	[0.3468, 0.3680]
	CO → PEB	Direct effect	−0.0093	0.0118	0.427	[−0.0324, 0.0137]
	CO → M → PEB	Total indirect	0.3667	0.0118	–	[0.3440, 0.3904]
EGM	EGM → PEB	Total effect	0.6118	0.0083	< 0.001	[0.5955, 0.6281]
	EGM → PEB	Direct effect	−0.0194	0.0199	0.329	[−0.0585, 0.0196]
	EGM → M → PEB	Total indirect	0.6312	0.0199	–	[0.5920, 0.6709]

Similarly, the total effect of EGM on PEB was significant (β = 0.6118, *p* < 0.001). After controlling for all mediating variables, its direct effect also became non-significant. This indicates that the mediating variables played a role of full mediation for both the CO → PEB and EGM → PEB relationships.

## 5 Discussion

This study first established the measurement model's validity, with a particular focus on discriminant validity using the rigorous HTMT criterion. The results confirmed that all HTMT values were well below the recommended threshold of 0.85, demonstrating that the seven concepts employed in this study are well-distinguished—representing seven independent concepts rather than overlapping constructs. This outcome aligns with modern psychometric standards, confirming that our questionnaire clearly and accurately measures distinct variables. This rigorous measurement foundation provides crucial assurance for subsequent analyses of inter-variable relationships. Previous research emphasizes that exploring causal mechanisms between variables only becomes persuasive when each variable is independently and accurately measured. Our findings reveal that while these variables are independent, they exhibit significant positive correlations, preliminarily supporting their potential existence within a continuous causal chain. Thus, strong discriminant validity enhances the reliability and credibility of our subsequent examination of the pathway “How VES influences PEB through psychological mechanisms.” This establishes a solid foundation for deepening our understanding of the complex processes through which virtual games impact real-world behavior.

Through descriptive statistics and correlation analysis, this study reveals that the mean values of all core variables fall within the upper-middle range. His positive reception is particularly noteworthy given our sample, which consists of highly engaged Honor of Kings players. Their deep immersion and significant time investment in the game may foster a stronger sense of community and loyalty, making them more receptive to the platform's pro-social messaging compared to casual players. The strong positive correlations found between in-game stimuli (CO and EGM) and players' psychological states (GAER and PET) are therefore not just a validation of the S-O-R framework's “S” influencing “O” premise (Ngo et al., 2024). They also reflect the unique context of a culturally dominant game like Honor of Kings, where in-game elements can become highly salient and influential for its dedicated player base.

Concurrently, intrinsic psychological states (e.g., GAER and PET) exhibit strong correlations with subsequent behavioral intentions and actual PEB, aligning with prior research concluding that psychological variables serve as critical antecedents to behavior (Ruan et al., 2022). These significant and consistent correlation patterns provide crucial preliminary evidence for subsequent testing of more complex chained mediation models. Previous research indicates that robust correlations among variables are a necessary prerequisite for the existence of underlying causal pathways. Therefore, these findings preliminarily outline a continuous pathway from game reception to real-world action, supporting the feasibility of exploring deeper underlying mechanisms.

This study employs chain mediation analysis to clearly reveal a complete psychological transformation pathway from VES to real-world PEB. Findings indicate that environmental design elements in games (CO and EGM) effectively stimulate players' emotional resonance, which primarily influences final PEB through two key pathways: first, by enhancing PET to strengthen environmental purchasing intent; second, by promoting social sharing of environmental information through PET. Notably, PET serves as a core bridging variable in both pathways, with the effect being most pronounced along the “GAER → PET → ITS → PEB” pathway, with the effect being most pronounced along the “GAER → PET → ITS → PEB” pathway (indirect effect = 0.390).

Furthermore, to construct a more comprehensive model within the S-O-R framework and compare direct effects with mediating effects, this study specifically examined the direct path from S to response R. After incorporating all mediating variables (GAER, PET, ITS, ITP) into the regression model, the direct effects of both CO (β = −0.0093, *p* = 0.427) and EGM (β = −0.0194, *p* = 0.329) on PEB became non-significant, while their total indirect effects remained significant. This result indicates that the identified psychological mechanisms (GAER, PET, ITP, ITS) fully mediate the relationship between in-game environmental stimuli and real-world pro-environmental behavior. This pattern of full mediation reinforces the conclusion that the influence of virtual environmental cues on behavior is not direct but is entirely transmitted through the sequential internal states of emotional response and trust, thereby validating the core premise of the S-O-R model in this context.

This finding aligns strongly with prior research exploring the complexity of psychological mechanisms. Previous studies consistently indicate that the conversion from cognitive emotions to actual actions is rarely a simple direct effect but relies on sequential transmission through a series of mediating variables (Asif et al., 2023; Li et al., 2021). The pivotal role of PET in this study corroborates this perspective, demonstrating that players' trust in the game platform's environmental commitments serves as the psychological foundation for transforming transient in-game emotional engagement into stable and transferable real-world behavioral intentions. This aligns with prior findings that trust plays a stabilizing and catalytic role in the attitude-behavior relationship (Krabbendam et al., 2024). A crucial point of contrast, however, lies in the nature of trust formation. Unlike trust in traditional, one-way media, trust within a gaming environment is forged through active participation and relational bonding. Games can foster a strong sense of brand community and even parasocial relationships with the game world, creating a deeper, more personal form of trust that makes players more receptive to the platform's messaging (Chen et al., 2025). Specifically, for a game like Honor of Kings, which is deeply integrated with social platforms (like WeChat and QQ) and fosters strong team-based gameplay, this trust is not merely transactional but communal. Players build relationships not just with the game's content, but with each other through the platform. This creates a strong sense of brand community and even parasocial relationships with the game world, fostering a deeper, more personal form of trust that makes players more receptive to the platform's pro-social messaging.

Moreover, our findings significantly extend the Stimulus-Organism-Response (S-O-R) framework by unpacking the ‘black box' of the Organism (O). Our model identifies two sequential internal states: an immediate affective reaction (GAER) followed by a more stable, cognitive judgment (PET). This emotional-cognitive sequence can be further illuminated by narrative transportation theory. The rich lore and world-building of Honor of Kings (S) facilitate player immersion, temporarily suspending real-world beliefs and making adolescents more susceptible to its embedded environmental themes. This emotional resonance (GAER) is a necessary precondition, but it is the subsequent cognitive formation of trust (PET) that solidifies this transient state into a durable intention.

Furthermore, the dual-path mechanism (purchasing and social sharing) identified in this study holds significant theoretical value. While prior research often focused on single behavioral dimensions, our findings demonstrate that virtual game incentives can simultaneously target both private and public spheres of PEB. More importantly, the social sharing pathway (ITS) exhibited the strongest effect. This finding can be interpreted through the lens of Social Identity Theory within the S-O-R framework. For deeply engaged players, the game community constitutes a salient in-group. Spreading environmental messages (R) serves as an “expressive” public behavior that allows players to signal their alignment with the pro-environmental values of this trusted community, thereby strengthening their social identity within it. In contrast, purchasing is a more private, “instrumental” behavior. This explains why the path through social sharing is more potent—it satisfies the fundamental need for social belonging, a powerful driver rooted in the Organism's social-cognitive processes.

Nonetheless, it is important to acknowledge the limitations of this study, which also open avenues for future research. A primary consideration is that all data were collected from a single source through self-report questionnaires completed by the players. This reliance on self-reported data, while effective for capturing subjective experiences, means that the observed relationships are based on player perceptions and could be influenced by factors such as social desirability. Although we implemented procedural measures like ensuring anonymity to minimize such effects, future research could build upon our findings with alternative methodologies. For instance, a longitudinal study would offer stronger evidence for the causal pathways we identified by tracking changes over time. Furthermore, incorporating multi-source data or employing objective measurements would help to more robustly validate the real-world behavioral outcomes.

## 6 Conclusion

This study's findings demonstrate a complete psychological conversion pathway: Virtual Environmental Stimuli (VES) within games, such as Character Outfits (CO) and Environmental Game Mechanisms (EGM), significantly stimulate adolescents' Game Appraisal and Emotional Response (GAER). This emotional and cognitive response, in turn, promotes real-world Pro-Environmental Behavior (PEB) by enhancing Pro-Environmental Intention (PEI).

Theoretical Implications The primary theoretical contribution of this research is the empirical identification of Pro-Environmental Trust (PET) as the crucial psychological bridge in this process. Our findings elucidate that PET is the core mechanism that transforms transient, situational in-game emotional resonance (GAER) into stable, transferable real-world behavioral intentions (PEI), namely the Intention to Purchase Environmental Products (ITP) and the Intention to Spread Environmental Messages (ITS). This study contributes to the literature by specifying how virtual engagement translates into real-world action, confirming that trust in the platform's environmental commitment serves as the psychological foundation for this behavioral conversion.

Practical Implications These findings offer focused, actionable implications for game developers and policymakers.

1. Prioritize Trust-Building: To effectively leverage games for environmental promotion, designing immersive content (CO and EGM) to elicit strong emotional responses (GAER) is insufficient on its own. Developers must strategically and equally prioritize building and maintaining player trust (PET) in the platform's long-term environmental commitments.
2. Target Diversified Behaviors: This research provides an empirically supported strategy for gamified interventions that can stimulate diversified forms of PEB, moving beyond the limits of traditional education. Game mechanics can be designed to simultaneously encourage private-sphere consumption behaviors (ITP) and public-sphere advocacy actions (ITS).
3. Leverage Social Advocacy: The psychological pathway culminating in social sharing (GAER → PET → ITS → PEB) exhibited the strongest overall effect size. This provides a clear directive: interventions should strongly leverage the social and community features inherent in gaming platforms to encourage peer-to-peer environmental communication, as this appears to be a particularly powerful driver of real-world behavioral change.

## Data Availability

The original contributions presented in the study are included in the article/Supplementary material, further inquiries can be directed to the corresponding author.
